# Supplementary material for: Leptospirosis Risk Assessment in Rodent Populations and Environmental Reservoirs in Humanitarian Aid Settings in Thailand
Source: Microorganisms. 2024 Dec 27;13(1):29. doi: 10.3390/microorganisms13010029 (PMC11767840; doi:10.3390/microorganisms13010029)
Supplement: Supplementary file 1 [file microorganisms-13-00029-s001.zip › microorganisms-3366569-supplementary.pdf]

Supplementary Materials:

Table S1: Number of Trapped Rodents at Pre-Exercise Leptospirosis Surveillance Sites in 15 Provinces (CBG Training Areas, 2017-2022)

Table S2: Trapping Success of Small Mammals by Surveillance Session in Pre-Exercise Leptospirosis Surveillance at CBG Training Sites

Table S3: Number, Percentage of Trapped Small Mammals, and Leptospirosis Prevalence by Species during Pre-Exercise Surveillance in CBG Training Sites (2017-2022)

Table S4: Distribution of Small Mammal Species in CBG Training Sites across 15 Leptospirosis Surveillance Provinces

Table S5: Geographical Distribution of *Leptospira* Species and STs in Positive lipL32 Rodents from CBG Training Areas (2017-2022)

Table S6: *Leptospira* Persistence in Environmental Reservoirs (Surveillance Years: 2017-2022)

Table S7: *Leptospira* Persistence and Natural Circulation in Water from Activity Sites Across 15 Provinces (Surveillance Years: 2017-2022)

Table S8: *Leptospira* Persistence and Natural Circulation in Soil from Activity Sites across Six Provinces (2021-2022)

Table S9: GenBank Accession Numbers of 16S rRNA Genes

Table S10: Accession Numbers of Housekeeping Genes in Multilocus Sequence Typing Scheme 1 Deposited in GenBank

Supplementary Materials: Table S1.

Table S1: Number of Trapped Rodents at Pre-Exercise Leptospirosis Surveillance Sites in 15 Provinces (CBG Training Areas, 2017-2022)

Provinces	CBG 17	CBG 18	CBG 19	CBG 20	CBG 21	CBG 22	No. of trapped rodents	% trapped rodents
Chachoengsao (CCS)	-	9	-	-	-	-	9	0.8
Chaiyaphum (CYP)	13	-	-	-	-	-	13	1.1
Chantaburi (CTB)	6	51	37	38	28	32	192	16.5
KhonKhan (KHK)	14	-	-	-	-	-	14	1.2
Krabi (KRB)	-	-	-	-	-	66	66	5.7
Lopburi (LBR)	-	54	-	-	-	-	54	4.7
Nakhon Ratchasima (NRS)	-	18	-	-	-	-	18	1.6
Nakhonsawan (NSW)	-	-	28	-	-	-	28	2.4
Phitsanulok (PSL)	-	-	14	102	-	-	116	10.0
Rayong (RAY)	3	23	47	83	202	19	337	32.5
SaKaeo (SKA)	-	-	-	-	18	-	18	1.6
Saraburi (SRB)	-	-	-	-	-	22	22	1.9
Sukhothai (SKT)	-	-	-	16	-	-	16	1.4
Tak (TAK)	-	-	83	-	-	-	83	7.1
Trat (TRA)	-	-	-	-	45	90	135	11.6
Total	36	115	209	239	293	229	1,161	100

Supplementary Materials: Table S2.

Table S2: Trapping Success of Small Mammals by Surveillance Session in Pre-Exercise Leptospirosis Surveillance at CBG Training Sites

CBG	Provinces	Number of traps night	Number of trapped rodents	Trapped success
**17**	Chaiyaphum, Chantaburi, KhonKhan, Rayong	8	36	4.5
**18**	Chachongsao, Chanthaburi, Lopburi, Nakhon Ratchasima, Rayong	12	155	12.9
**19**	Chantaburi, Nakhonsawan, Phitsanulok, Rayong, Tak	17	209	12.3
**20**	Chantaburi, Phitsanulok, Rayong, Sukhothai	15	239	15.9
**21**	Chantaburi, Rayong, SaKaeo, Trat	18	293	16.3
**22**	Chantaburi, Krabi, Rayong, Saraburi, Trat	21	229	10.9

Supplementary Materials: Table S3.

Table S3: Number, Percentage of Trapped Small Mammals, and Leptospirosis Prevalence by Species during Pre-Exercise Surveillance in CBG Training Sites (2017-2022)

Host Species	CBG 17	CBG 18	CBG 19	CBG 20	CBG 21	CBG 22	Total	% Trapped small mammals	% Infected rodents by species	% Infection across all trapped rodents
Bandicota indica (BAIN)	5	44	52	63	9	6	179	15.4	1.7	0.3
Bandicota savilei (BASA)	1	50	48	78	14	1	192	16.5	1.0	0.2
Berylmys berdmorei (BEBE)	1	2	13	7	5	-	28	2.4	7.1	0.2
Berylmys bowersi (BEBO)	-	-	-	1	-	-	1	0.1	na	na
Maxomy surifer (MASU)	-	-	18	-	1	-	19	1.6	na	na
Menetes berdmori (MEBE)	2	2	7	-	1	1	13	1.1	na	na
Mus caroli (MUCA)	1	1	1	-	1	4	8	0.7	na	na
Mus cervicolor (MUCE)	-	4	1	-	1	-	6	0.5	16.7	0.1
Niviventer fulvescens (NIFU)	-	1	4	1	-	-	6	0.5	na	na
Rattus exulans (RAEX)	-	6	38	23	13	6	86	7.4	na	na
Rattus norvegicus (RANO)	1	-	3	-	-	1	5	0.4	na	na
Rattus rattus (RATA)	25	45	17	66	248	196	597	51.4	1.2	0.6
Tupaia belangeri (TUBE)	-	-	7	-	-	5	12	1.0	na	na
Tupaia glis (TUGL)	-	-	-	-	-	9	9	0.8	na	na
**% Infected rodents by CBG session**	**0.0**	**1.3**	**1.0**	**1.3**	**0.7**	**2.6**	**1.3**	-	-	-
**Total**	**36**	**155**	**209**	**239**	**293**	**229**	**1161**	**100.0**	**1.3**	**1.3**

Supplementary Materials: Table S4.

Table S4: Distribution of Small Mammal Species in CBG Training Sites across 15 Leptospirosis Surveillance Provinces

**Province**	**Trapped rodent species**														
	BAIN	***BASA***	***BEBE***	***BEBO***	***MASU***	***MEBE***	***MUCA***	***MUCE***	***NIFU***	***RAEX***	***RANO***	***RATA***	***TUBE***	***TUGL***	***Total***
Chachoengsao (CCS)	3	3	-	-	-	2	-	-	-	1	-	-	-	-	**9**
Chaiyaphum (CYP)	-	-	-	-	-	-	-	-	-	-	-	13	-	-	**13**
Chantaburi (CTB)	29	37	12	-	-	2	-	-	1	24	1	86	-	-	**192**
KhonKhan (KHK)	1	-	-	-	-	-	-	-	-	-	1	12	-	-	**14**
Krabi (KRB)	3	-	-	-	-	-	-	-	-	-	-	50	5	8	**66**
Lopburi (LBR)	22	23	-	-	-	-	-	-	-	2	-	7	-	-	**54**
Nakhon Ratchasima (NRS)	7	-	-	-	-	-	1	4	-	2	-	4	-	-	**18**
Nakhonsawan (NSW)	4	-	-	-	-	-	-	1	-	19	3	1	-	-	**28**
Phitsanulok (PSL)	39	48	-	-	-	2	-	-	-	16	-	9	2	-	**116**
Rayong (RAY)	28	24	16	1	19	6	2	-	5	14	-	261	1	-	**377**
SaKaeo (SKA)	-	11	-	-	-	1	-	1	-	-	-	5	-	-	**18**
Saraburi (SRB)	1	-	-	-	-	-	4	-	-	2	-	14	-	1	**22**
Sukhothai (SKT)	2	8	-	-	-	-	-	-	-	5	-	1	-	-	**16**
Tak (TAK)	38	38	-	-	-	-	1	-	-	-	-	2	4	-	**83**
Trat (TRA)	2	-	-	-	-	-	-	-	-	1	-	132	-	-	**135**
**Total**	**179**	**192**	**28**	**1**	**19**	**13**	**8**	**6**	**6**	**86**	**5**	**597**	**12**	**9**	**1,161**

Supplementary Materials: Table S5.

Table S5: Geographical Distribution of *Leptospira* Species and STs in Positive lipL32 Rodents from CBG Training Areas (2017-2022)

Province	Sample no.	*Leptospira* spp.	Host species	CBG	No. of loci amplification	ST
Chantaburi	DS3704	*L. interrogans*	*Rattus rattus*	18	5 of 7	na
	MS1414	*L. interrogans*	*Rattus rattus*	20	0 of 7	na
	MS1417	*L. interrogans*	*Berylmys berdmorei*	20	7 of 7	341
	MS1419	*L. interrogans*	*Berylmys berdmorei*	20	7 of 7	342
	MS2876	*L. interrogans*	*Bandicota savilei*	21	7 of 7	34
Krabi	MS3394	*L. interrogans*	*Rattus rattus*	22	7 of 7	205
	MS3409	*L. interrogans*	*Rattus rattus*	22	7 of 7	205
	MS3427	*L. interrogans*	*Rattus rattus*	22	7 of 7	205
	MS3428	*L. interrogans*	*Bandicota indica*	22	7 of 7	205
	MS3429	*L. interrogans*	*Bandicota indica*	22	0 of 7	na
Nakhon Ratchasima	DS3574	*L. borgpetersenii*	*Mus cervicolor*	18	7 of 7	259
Rayong	MS2764	*L. interrogans*	*Rattus rattus*	21	7 of 7	235
Tak	DS5249	*L. borgpetersenii*	*Bandicota indica*	19	0 of 7	na
	DS5256	*L. interrogans*	*Bandicota savilei*	19	7 of 7	34
Trat	MS3217	*L. interrogans*	*Rattus rattus*	22	7 of 7	337

Supplementary Materials: Table S6.

Table S6: *Leptospira* Persistence in Environmental Reservoirs (Surveillance Years: 2017-2022[a])

Environmental Sources	CBG FY	Number of collected samples	*Leptospira* Q -PCR positive (% persistence)	*Leptospira* culture positive (% recovery)
Water	17	36	0 (0%)	3 (8.3%)
	18	105	25 (23.8%)	8 (7.6%)
	19	102	8 (7.8%)	7 (6.9%)
	20	57	6 (10.5%)	4 (7.0%)
	21	63	1 (1.6%)	2 (3.2%)
	22	50	2 (4%)	3 (6.0%)
Total		413	42 (10.2%)	27 (6.5%)
Soil[a]	21	61	13 (21.3%)	28 (46.0%)
	22	69	17 (24.6%)	12 (17.4%)
Total		130	30 (23.1%)	40 (30.8%)

Note:

a) **Pre-surveillance of** *Leptospira* persistence and isolate recovery in soil were only assessed for CBG21 and CBG22.

Supplementary Materials: Table S7.

Table S7: *Leptospira* Persistence and Natural Circulation in Water from Activity Sites Across 15 Provinces (Surveillance Years: 2017-2022)

CBG FY	Province	No. of collected samples	No. of *Leptospira* positive [a] (% persistence)	No. of isolated *Leptospira* [b] (% recovery)
17				
	Chantaburi	10	0	0
	Chaiyaphum	9	0	2 (22%)
	Khonkhan	7	0	1 (14%)
	Rayong	10	0	0
18				
	Chachoengsao	22	2 (9%)	1 (5%)
	Chantaburi	20	5 (25%)	2 (10%)
	Lopburi	21	6 (29%)	1 (5%)
	Nakhon Ratchasima	22	3 (14%)	2 (9%)
	Rayong	20	9 (45%)	2 (10%)
19				
	Chantaburi	13	2 (15%)	1 (8%)
	Nakhonsawan	20	4 (20%)	3 (15%)
	Phitsanulok	20	0	1 (5%)
	Rayong	20	0	0
	Tak	29	2 (7%)	2 (7%)
20				
	Chantaburi	10	3 (30%)	0
	Phitsanulok	25	0	3 (12%)
	Rayong	11	3 (27%)	1 (9%)
	Sukhothai	11	0	0
21				
	Chantaburi	12	0	0
	Rayong	27	0	0
	SaKaeo	12	1 (8%)	2 (17%)
	Trat	12	0	0
22				
	Chantaburi	10	0	0
	Krabi	10	0	0
	Rayong	10	1 (10%)	2 (20%)
	Saraburi	10	1 (10%)	1 (10%)
	Trat	10	0	0

Note:

a) *Leptospira* is identified in water samples to determine its persistence.

b) *Leptospira* isolates recovered from water samples indicate natural circulation.

Supplementary Materials: Table S8

Table S8: *Leptospira* Persistence and Natural Circulation in Soil from Activity Sites across Six Provinces (2021-2022)

CBG FY	Province	No. of collected samples	No. of *Leptospira* positive [a] (% persistence)	No. of isolated *Leptospira* [b] (% recovery)
21				
	Chantaburi	8	2 (25%)	5 (63%)
	Rayong	37	11 (30%)	16 (43%)
	SaKaeo	7	0	5 (71%)
	Trat	9	0	2 (22%)
22				
	Chantaburi	14	6 (43%)	5 (36%)
	Krabi	14	4 (29%)	1 (7%)
	Rayong	14	3 (21%)	4 (29%)
	Saraburi	10	1 (10%)	1 (10%)
	Trat	17	3 (18%)	1 (6%)

Note:

a) *Leptospira* is identified in soil samples to determine its persistence.
b) *Leptospira* isolates recovered from soil samples indicate natural circulation.

Supplementary Materials: Table S9:

Table S9: GenBank Accession Numbers of 16S rRNA Genes

This table presents GenBank accession numbers for 16S rRNA gene sequences from two sources: positive *Leptospira* screening (P: partial 16SrRNA gene) and isolated *Leptospira* (W: near complete of 16SrRNA gene). It also includes reference sequences [1] for *Leptospira* spp. used in phylogenetic analysis.

**Groups**	**Species**	**Serovar**	**Strain**	**GenBank ID**	**Sources**	**ID**
Pathogen	*L. borgpetersenii*	Javanica	Veldrat Batavia 46	NR_043259.1	REFERENCE	*L. borgpetersenii* NR043259.1
Pathogen	*L. borgpetersenii*	Javanica	Veldrat Batavia 46	NR_114969.1	REFERENCE	*L. borgpetersenii* NR114969.1
Pathogen	*L. interrogans*	Pyrogenes	L0374	EF536990.1	REFERENCE	*L. interrogans* EF536990.1
Pathogen	*L. interrogans*	Undesignated	UT364	EF536981.1	REFERENCE	*L. interrogans* EF536981.1
Pathogen	*L. interrogans*	Grippotyphosa	L1006	EF536975.1	REFERENCE	*L. interrogans* EF536975.1
Pathogen	*L. interrogans*	Autumnalis	L0013	EF536978.1	REFERENCE	*L. interrogans* EF536978.1
Pathogen	*L. interrogans*	Bataviae	UT075	EF536993.1	REFERENCE	*L. interrogans* EF536993.1
Pathogen	*L. interrogans*	Undesignated	RGA	NR_029361.1	REFERENCE	*L. interrogans* NR029361.1
Pathogen	*L. interrogans*	Icterohaemorrhagiae	RGA	NR_114968.1	REFERENCE	*L. interrogans* NR114968.1
Pathogen	*L. interrogans*	Undesignated	RGA	NR_115234.1	REFERENCE	*L. interrogans* NR115234.1
Pathogen	*L. interrogans*	Icterohaemorrhagiae	RGA	NR_116542.1	REFERENCE	*L. interrogans* NR116542.1
Pathogen	*L. kmetyi*	Malaysia	Bejo-Iso9	AB279549.1	REFERENCE	*L. kmetyi* AB279549.1
Pathogen	*L. kmetyi*	Malaysia	Bejo-Iso9	NR_041544.1	REFERENCE	*L. kmetyi* NR041544.1
Pathogen	*L. yasudae*	Undesignated	CES	MN060986.1	REFERENCE	*L. yasudae* MN060986.1
Pathogen	*L. yasudae*	Undesignated	F1	NR_180240.1	REFERENCE	*L. yasudae* NR180240.1
Intermediate	*L. andrefontaineae*	Undesignated	CES	MN062722.1	REFERENCE	*L. andrefontaineae* MN062722.1
Intermediate	*L. dzoumogneensis*	Undesignated	CES	MN060997.1	REFERENCE	*L. dzoumogneensis* MN060997.1
Intermediate	*L. hartskeerlii*	Undesignated	CES	MK791631.1	REFERENCE	*L. hartskeerlii* MK791631.1
Intermediate	*L. johnsonii*	Undesignated	CES	MN062721.1	REFERENCE	*L. johnsonii* MN062721.1
Intermediate	*L. koniambonensis*	Undesignated	CES	MN061003.1	REFERENCE	*L. koniambonensis* MN061003.1
Intermediate	*L. licerasiae*	Varillal	VAR 010	NR_044310.1	REFERENCE	*L. licerasiae* NR044310.1
Intermediate	*L. licerasiae*	Varillal	CEH033	EF612280.1	REFERENCE	*L. licerasiae* EF612280.1

Intermediate	*L. licerasiae*	Undesignated	CES	MK791628.1	REFERENCE	*L. licerasiae* MK791628.1
Intermediate	*L. perolatii*	Undesignated	CES	MK791629.1	REFERENCE	*L. perolatii* MK791629.1
Intermediate	*L. selangorensis*	Undesignated	CES	MN062732.1	REFERENCE	*L. selangorensis* MN062732.1
Intermediate	*L. wolffii*	Undesignated	CES	MK791626.1	REFERENCE	*L. wolffii* MK791626.1
Intermediate	*L. wolffii*	Undesignated	LS0914U	KC662454.1	REFERENCE	*L. wolffii* KC662454.1
Saprophyte	*L. bandrabouensis*	Undesignated	CES	MN062727.1	REFERENCE	*L. bandrabouensis* MN062727.1
Saprophyte	*L. biflexa*	Patoc	Patoc 1	NR_043043.1	REFERENCE	*L. biflexa* NR043043.1
Saprophyte	*L. idonii*	Undesignated	CES	MN086352.1	REFERENCE	*L. idonii* MN086352.1
Saprophyte	*L. jelokensis*	Undesignated	CES	MN062728.1	REFERENCE	*L. jelokensis* MN062728.1
Saprophyte	*L. kemamanensis*	Undesignated	CES	MN062726.1	REFERENCE	*L. kemamanensis* MN062726.1
Saprophyte	*L. macculloughii*	Undesignated	CES	MK791638.1	REFERENCE	*L. macculloughii* MK791638.1
Saprophyte	*L. montravelensis*	Undesignated	CES	MN062731.1	REFERENCE	*L. montravelensis* MN062731.1
Saprophyte	*L. mtsangambouensis*	Undesignated	CES	MN062733.1	REFERENCE	*L. mtsangambouensis* MN062733.1
Saprophyte	*L. terpstrae*	Undesignated	Hualin	NR_115294.1	REFERENCE	*L. terpstrae* NR_115294.1
Saprophyte	*L. wolbachii*	Codice	CDC	NR_043046.1	REFERENCE	*L. wolbachii* NR043046.1
Saprophyte	*L. yanagawae*	Saopaulo	Sao Paulo	NR_115293.1	REFERENCE	*L. yanagawae* NR_115293.1
Other genus in Leptospiraceae	*Leptonema illini*	Undesignated	3055	NR_043139.1	REFERENCE	*Leptonema* NR043139.1
Pathogen	*L. interrogans*	Undesignated	DS3704	PP823824	Rodent kidney_P	DS3704 RATA CTB
Pathogen	*L. interrogans*	Undesignated	MS1414	PP823827	Rodent kidney_P	MS1414 RATA CTB
Pathogen	*L. interrogans*	Undesignated	MS1417	PP823828	Rodent kidney_P	MS1417 BEBE CTB
Pathogen	*L. interrogans*	Undesignated	MS1419	PP823829	Rodent kidney_P	MS1419 BEBE CTB
Pathogen	*L. interrogans*	Undesignated	MS2876	PP823831	Rodent kidney_P	MS2876 BASA CTB
Pathogen	*L. interrogans*	Undesignated	MS3394	PP823833	Rodent kidney_P	MS3394 RATA KRB
Pathogen	*L. interrogans*	Undesignated	MS3409	PP823834	Rodent kidney_P	MS3409 RATA KRB
Pathogen	*L. interrogans*	Undesignated	MS3427	PP823835	Rodent kidney_P	MS3427 RATA KRB
Pathogen	*L. interrogans*	Undesignated	MS3428	PP823836	Rodent kidney_P	MS3428 BAIN KRB
Pathogen	*L. interrogans*	Undesignated	MS3429	PP823837	Rodent kidney_P	MS3429 BAIN KRB
Pathogen	*L. borgpetersenii*	Undesignated	DS3574	PP823823	Rodent kidney_P	DS3574 MUCE NRS
Pathogen	*L. interrogans*	Undesignated	MS2764	PP823830	Rodent kidney_P	MS2764 RATA RAY

Pathogen	*L. borgpetersenii*	Undesignated	DS5249	PP823825	Rodent kidney_P	DS5249 BAIN TAK
Pathogen	*L. interrogans*	Undesignated	DS5256	PP823826	Rodent kidney_P	DS5256 BASA TAK
Pathogen	*L. interrogans*	Undesignated	MS3217	PP823832	Rodent kidney_P	MS3217 RATA TRA
Pathogen	*L. interrogans*	Undesignated	MS1416U	PP818988	Rodent urine_W	MS1416U BAIN CTB
Pathogen	*L. interrogans*	Undesignated	MS2758	PP818989	Rodent kidney_W	MS2758 RATA RAY
Pathogen	*L. interrogans*	Undesignated	MS3217U	PP818990	Rodent urine_W	MS3217U RATA TRA
Pathogen	*L. interrogans*	Undesignated	MS3265U	PP818991	Rodent urine_W	MS3265U BAIN TRA
Pathogen	*L. interrogans*	Undesignated	WSCB102	PP819165	Water_P	WSCB102 CTB
Pathogen	*L. yasudae*	Undesignated	WSCB149	PP819179	Water_P	WSCB149 NSW
Pathogen	*L. yasudae*	Undesignated	WSCB399	PP819193	Water_P	WSCB399 RAY
Pathogen	*L. yasudae*	Undesignated	WSCB364	PP819192	Water_P	WSCB364 SRB
Pathogen	*L. yasudae*	Undesignated	WSCB141	PP819177	Water_P	WSCB141 RAY
Pathogen	*L. yasudae*	Undesignated	WSCB137	PP819176	Water_P	WSCB137 RAY
Pathogen	*L. yasudae*	Undesignated	WSCB123	PP819170	Water_P	WSCB123 RAY
Pathogen	*L. yasudae*	Undesignated	WSCB073	PP819158	Water_P	WSCB073 CCS
Pathogen	*L. yasudae*	Undesignated	WSCB057	PP819156	Water_P	WSCB057 NRS
Intermediate	*L. wolffii*	Undesignated	WSCB047	PP819154	Water_P	WSCB047 NRS
Intermediate	*L. wolffii*	Undesignated	WSCB048	PP819155	Water_P	WSCB048 NRS
Intermediate	*L. wolffii*	Undesignated	WSCB094	PP819163	Water_P	WSCB094 LBR
Intermediate	*L. wolffii*	Undesignated	WSCB131	PP819172	Water_P	WSCB131 RAY
Intermediate	*L. wolffii*	Undesignated	WSCB132	PP819173	Water_P	WSCB132 RAY
Intermediate	*L. yanagawae*	Undesignated	WSCB202	PP819182	Water_P	WSCB202 CTB
Intermediate	*L. wolffii*	Undesignated	WSCB227	PP819183	Water_P	WSCB227 TAK
Intermediate	*L. wolffii*	Undesignated	WSCB247	PP819185	Water_P	WSCB247 CTB
Intermediate	*L. wolffii*	Undesignated	WSCB249	PP819186	Water_P	WSCB249 CTB
Intermediate	*L. wolffii*	Undesignated	WSCB251	PP819187	Water_P	WSCB251 CTB
Intermediate	*L. wolffii*	Undesignated	WSCB255	PP819188	Water_P	WSCB255 RAY
Intermediate	*L. wolffii*	Undesignated	WSCB355	PP819191	Water_P	WSCB355 SKA
Intermediate	*L. wolffii*	Undesignated	WSCB260	PP819189	Water_P	WSCB260 RAY

Intermediate	*L. wolffii*	Undesignated	WSCB095	PP819164	Water_P	WSCB095 LBR
Intermediate	*L. wolffii*	Undesignated	WSCB154	PP819181	Water_P	WSCB154 NSW
Intermediate	*L. licerasiae*	Undesignated	WSCB242	PP819184	Water_P	WSCB242 TAK
Intermediate	*L. licerasiae*	Undesignated	WSCB133	PP819174	Water_P	WSCB133 RAY
Intermediate	*L. licerasiae*	Undesignated	WSCB111	PP819169	Water_P	WSCB111 CTB
Intermediate	*L. licerasiae*	Undesignated	WSCB108	PP819168	Water_P	WSCB108 CTB
Intermediate	*L. licerasiae*	Undesignated	WSCB092	PP819162	Water_P	WSCB092 LBR
Intermediate	*L. licerasiae*	Undesignated	WSCB087	PP819160	Water_P	WSCB087 LBR
Intermediate	*L. perolatii*	Undesignated	WSCB065	PP819157	Water_P	WSCB065 CCS
Pathogen	*L. yasudae*	Undesignated	WSCB137	PP819131	Water_W	WSCB137 RAY
Pathogen	*L. yasudae*	Undesignated	WSCB141	PP819132	Water_W	WSCB141 RAY
Pathogen	*L. yasudae*	Undesignated	WSCB399	PP819147	Water_W	WSCB399 RAY
Pathogen	*L. kmetyi*	Undesignated	WSCB355C2	PP819145	Water_W	WSCB355C2 SKA
Intermediate	*L. licerasiae*	Undesignated	WSCB47	PP819125	Water_W	WSCB47 NRS
Intermediate	*L. licerasiae*	Undesignated	WSCB355C1	PP819144	Water_W	WSCB355C1 SKA
Intermediate	*L. koniamboensis*	Undesignated	WSCB240	PP819138	Water_W	WSCB240 TAK
Intermediate	*L. hartskeerlii*	Undesignated	WSCB242	PP819139	Water_W	WSCB242 TAK
Intermediate	*L. wolffii*	Undesignated	WSCB142	PP819133	Water_W	WSCB142 NSW
Intermediate	*L. wolffii*	Undesignated	WSCB150	PP819134	Water_W	WSCB150 NSW
Saprophyte	Unidentified *Leptospira*	Undesignated	WSCB25	PP819122	Water_W	WSCB25 CYP
Saprophyte	*L. mtsangambouensis*	Undesignated	WSCB285	PP819143	Water_W	WSCB285 PSL
Saprophyte	*L. mtsangambouensis*	Undesignated	WSCB202	PP819137	Water_W	WSCB202 CTB
Saprophyte	*L. mtsangambouensis*	Undesignated	WSCB403	PP819148	Water_W	WSCB403 RAY
Saprophyte	*L. mtsangambouensis*	Undesignated	WSCB28	PP819123	Water_W	WSCB28 CYP
Saprophyte	*L. mtsangambouensis*	Undesignated	WSCB86	PP819128	Water_W	WSCB86 LBR
Saprophyte	*L. mtsangambouensis*	Undesignated	WSCB372	PP819146	Water_W	WSCB372 SRB
Saprophyte	*L. mtsangambouensis*	Undesignated	WSCB30	PP819124	Water_W	WSCB30 KHK
Saprophyte	*L. mtsangambouensis*	Undesignated	WSCB48	PP819126	Water_W	WSCB48 NRS
Saprophyte	*L. montravelensis*	Undesignated	WSCB162	PP819135	Water_W	WSCB162 NSW

Saprophyte	*L. montravelensis*	Undesignated	WSCB170	PP819136	Water_W	WSCB170 PSL
Saprophyte	*L. kemamanensis*	Undesignated	WSCB66	PP819127	Water_W	WSCB66 CCS
Saprophyte	*L. kemamanensis*	Undesignated	WSCB112	PP819129	Water_W	WSCB112 CTB
Saprophyte	*L. kemamanensis*	Undesignated	WSCB113	PP819130	Water_W	WSCB113 CTB
Saprophyte	*L. kemamanensis*	Undesignated	WSCB280	PP819142	Water_W	WSCB280 PSL
Saprophyte	*L. kemamanensis*	Undesignated	WSCB263	PP819140	Water_W	WSCB263 RAY
Saprophyte	*L. kemamanensis*	Undesignated	WSCB279	PP819141	Water_W	WSCB279 PSL
Pathogen	*L. yasudae*	Undesignated	CBS103	PP819010	Soil_P	CBS103 RAY
Pathogen	*L. yasudae*	Undesignated	CBS095	PP819008	Soil_P	CBS095 TRA
Pathogen	*L. yasudae*	Undesignated	CBS022	PP818992	Soil_P	CBS022 CTB
Intermediate	Unidentified *Leptospira*	Undesignated	CBS042	PP818993	Soil_P	CBS042 RAY
Intermediate	Unidentified *Leptospira*	Undesignated	CBS043	PP818994	Soil_P	CBS043 RAY
Intermediate	Unidentified *Leptospira*	Undesignated	CBS049	PP818995	Soil_P	CBS049 RAY
Intermediate	Unidentified *Leptospira*	Undesignated	CBS051	PP818996	Soil_P	CBS051 RAY
Intermediate	Unidentified *Leptospira*	Undesignated	CBS052	PP818997	Soil_P	CBS052 RAY
Intermediate	Unidentified *Leptospira*	Undesignated	CBS054	PP818998	Soil_P	CBS054 RAY
Intermediate	Unidentified *Leptospira*	Undesignated	CBS055	PP818999	Soil_P	CBS055 RAY
Intermediate	Unidentified *Leptospira*	Undesignated	CBS056	PP819000	Soil_P	CBS056 RAY
Intermediate	Unidentified *Leptospira*	Undesignated	CBS061	PP819001	Soil_P	CBS061 RAY
Intermediate	Unidentified *Leptospira*	Undesignated	CBS066	PP819002	Soil_P	CBS066 SRB
Intermediate	Unidentified *Leptospira*	Undesignated	CBS074	PP819003	Soil_P	CBS074 KRB
Intermediate	Unidentified *Leptospira*	Undesignated	CBS083	PP819006	Soil_P	CBS083 KRB
Intermediate	Unidentified *Leptospira*	Undesignated	CBS087	PP819007	Soil_P	CBS087 TRA
Intermediate	Unidentified *Leptospira*	Undesignated	CBS101	PP819009	Soil_P	CBS101 TRA
Intermediate	Unidentified *Leptospira*	Undesignated	CBS107	PP819012	Soil_P	CBS107 RAY
Intermediate	Unidentified *Leptospira*	Undesignated	CBS117	PP819013	Soil_P	CBS117 CTB
Intermediate	Unidentified *Leptospira*	Undesignated	CBS129	PP819017	Soil_P	CBS129 CTB
Intermediate	Unidentified *Leptospira*	Undesignated	CBS130	PP819018	Soil_P	CBS130 CTB
Pathogen	*L. kmetyi*	Undesignated	CBS009	PP819078	Soil_W	CBS009 RAY

Pathogen	*L. yasudae*	Undesignated	CBS010	PP819079	Soil_W	CBS010 RAY
Pathogen	*L. yasudae*	Undesignated	CBS037	PP819094	Soil_W	CBS037 SKA
Pathogen	*L. yasudae*	Undesignated	CBS053	PP819098	Soil_W	CBS053 RAY
Pathogen	*L. yasudae*	Undesignated	CBS061	PP819101	Soil_W	CBS061 RAY
Intermediate	*L. wolffii*	Undesignated	CBS001	PP819074	Soil_W	CBS001 RAY
Intermediate	*L. wolffii*	Undesignated	CBS034	PP819091	Soil_W	CBS034 TRA
Intermediate	*L. dzoumogneensis*	Undesignated	CBS059	PP819100	Soil_W	CBS059 RAY
Intermediate	Unidentified *Leptospira*	Undesignated	CBS022	PP819087	Soil_W	CBS022 CTB
Intermediate	*L. johnsonii*	Undesignated	CBS049	PP819097	Soil_W	CBS049 RAY
Intermediate	Unidentified *Leptospira*	Undesignated	CBS038	PP819095	Soil_W	CBS038 SKA
Intermediate	Unidentified *Leptospira*	Undesignated	CBS039	PP819096	Soil_W	CBS039 SKA
Intermediate	Unidentified *Leptospira*	Undesignated	CBS111	PP819108	Soil_W	CBS111 RAY
Intermediate	Unidentified *Leptospira*	Undesignated	CBS069	PP819102	Soil_W	CBS069 SRB
Intermediate	*L. licerasiae*	Undesignated	CBS012	PP819081	Soil_W	CBS012 RAY
Saprophyte	*L. idonii*	Undesignated	CBS072	PP819103	Soil_W	CBS072 KRB
Saprophyte	*L. kemamanensis*	Undesignated	CBS015	PP819083	Soil_W	CBS015 RAY
Saprophyte	*L. mtsangambouensis*	Undesignated	CBS004	PP819075	Soil_W	CBS004 RAY
Saprophyte	*L. mtsangambouensis*	Undesignated	CBS011	PP819080	Soil_W	CBS011 RAY
Saprophyte	*L. mtsangambouensis*	Undesignated	CBS014	PP819082	Soil_W	CBS014 RAY
Saprophyte	*L. mtsangambouensis*	Undesignated	CBS017	PP819084	Soil_W	CBS017 RAY
Saprophyte	*L. mtsangambouensis*	Undesignated	CBS021	PP819086	Soil_W	CBS021 CTB
Saprophyte	*L. mtsangambouensis*	Undesignated	CBS023	PP819088	Soil_W	CBS023 CTB
Saprophyte	*L. mtsangambouensis*	Undesignated	CBS024	PP819089	Soil_W	CBS024 CTB
Saprophyte	*L. mtsangambouensis*	Undesignated	CBS032	PP819090	Soil_W	CBS032 TRA
Saprophyte	*L. mtsangambouensis*	Undesignated	CBS035	PP819092	Soil_W	CBS035 SKA
Saprophyte	*L. mtsangambouensis*	Undesignated	CBS055	PP819099	Soil_W	CBS055 RAY
Saprophyte	*L. mtsangambouensis*	Undesignated	CBS104	PP819105	Soil_W	CBS104 RAY
Saprophyte	*L. mtsangambouensis*	Undesignated	CBS107	PP819106	Soil_W	CBS107 RAY
Saprophyte	*L. mtsangambouensis*	Undesignated	CBS109	PP819107	Soil_W	CBS109 RAY

Saprophyte	*L. mtsangambouensis*	Undesignated	CBS117	PP819109	Soil_W	CBS117 CTB
Saprophyte	*L. mtsangambouensis*	Undesignated	CBS123	PP819110	Soil_W	CBS123 CTB
Saprophyte	*L. mtsangambouensis*	Undesignated	CBS124	PP819111	Soil_W	CBS124 CTB
Saprophyte	*L. mtsangambouensis*	Undesignated	CBS129	PP819113	Soil_W	CBS129 CTB
Saprophyte	*L. mtsangambouensis*	Undesignated	CBS005	PP819076	Soil_W	CBS005 RAY
Saprophyte	*L. mtsangambouensis*	Undesignated	CBS006	PP819077	Soil_W	CBS006 RAY
Saprophyte	*L. mtsangambouensis*	Undesignated	CBS020	PP819085	Soil_W	CBS020 CTB
Saprophyte	*L. mtsangambouensis*	Undesignated	CBS036	PP819093	Soil_W	CBS036 SKA
Saprophyte	*L. mtsangambouensis*	Undesignated	CBS095	PP819104	Soil_W	CBS095 TRA
Saprophyte	*L. mtsangambouensis*	Undesignated	CBS126	PP819112	Soil_W	CBS126 CTB

1. Benson, D.A., et al., *GenBank.* Nucleic Acids Res, 2014. **42**(Database issue): p. D32-7.

Supplementary Materials: Table S10.

Table S10: Accession Numbers of Housekeeping Genes in Multilocus Sequence Typing Scheme 1 Deposited in GenBank

ID	ST	Typing	glmU	pntA	sucA	tpiA	pfkB	mreA	caiB
DS3574 MUCE NRS	259	nMLST	PP834303	PP834314	PP834347	PP834358	PP834325	PP834336	PP874348
DS5256 BASA TAK	34	nMLST	PP834304	PP834315	PP834348	PP834359	PP834326	PP834337	PP874349
MS1417 BEBE CTB	341	nMLST	PP834305	PP834316	PP834349	PP834360	PP834327	PP834338	PP874350
MS1419 BEBE CTB	342	nMLST	PP834306	PP834317	PP834350	PP834361	PP834328	PP834339	PP874351
MS2764 RATA RAY	235	nMLST	PP834307	PP834318	PP834351	PP834362	PP834329	PP834340	PP874352
MS2876 BASA CTB	34	nMLST	PP834308	PP834319	PP834352	PP834363	PP834330	PP834341	PP874356
MS3217 RATA TRA	337	nMLST	PP834309	PP834320	PP834353	PP834364	PP834331	PP834342	PP874353
MS3394 RATA KRB	205	nMLST	PP834310	PP834321	PP834354	PP834365	PP834332	PP834343	PP874354
MS3409 RATA KRB	205	nMLST	PP834311	PP834322	PP834355	PP834366	PP834333	PP834344	PP874355
MS3427 RATA KRB	205	nMLST	PP834312	PP834323	PP834356	PP834367	PP834334	PP834345	PP874357
MS3428 BAIN KRB	205	nMLST	PP834313	PP834324	PP834357	PP834368	PP834335	PP834346	PP874358
MS1416U BAIN CTB	324	MLST	PP834451	PP834455	PP834471	PP834475	PP834459	PP834467	PP834463
MS2758 RATA RAY	49	MLST	PP834452	PP834456	PP834472	PP834476	PP834460	PP834468	PP834464
MS3217U RATA TRA	337	MLST	PP834453	PP834457	PP834473	PP834477	PP834461	PP834469	PP834465
MS3265U BAIN TRA	338	MLST	PP834454	PP834458	PP834474	PP834478	PP834462	PP834470	PP834466
